# Supplementary material for: Determining the perceptions and practices of oncologists regarding venous thromboembolism risk assessment in ambulatory cancer patients: A qualitative study
Source: PLoS One. 2025 Jan 6;20(1):e0316801. doi: 10.1371/journal.pone.0316801 (PMC11703059; doi:10.1371/journal.pone.0316801)
Supplement: S1 Appendix — (DOCX) [file pone.0316801.s001.docx]

**S1 Appendix: Interview Guide**

**Section A: Demographic Characteristics**

|  |
| --- |

1. What is your age?

| Male | □ |
| --- | --- |
| Female | □ |

1. Gender

|  |
| --- |

1. How many Years of experience in oncology do you have at the current hospital?
2. What is your academic degree?

| PH. D (board) | **□** |
| --- | --- |
| MSc | **□** |
| High diploma | **□** |

**Section B: The next set of questions are related to perceptions and beliefs**

1. How often do you detect VTE among your cancer patients? Probes: is VTE common? For which cancer patients it is more common?

2. How often do you conduct VTE risk assessment for your cancer patients?

3. How do you assess the risk of VTE for your cancer patients? If use specific scoring system, then use the following probes

a) Which one?

b) Why do you choose this score?

c) Can you describe how do you assess the VTE risk by this score?

d) What are the challenges for application of this score in daily practice?

e) What can influence your usage of such scoring system? (e.g., online calculator)

4. How easy or difficult would it be to conduct VTE risk assessment for your cancer patients? Probe: Is there any problems/difficulties do you think you might encounter in conducting VTE risk assessment for your patients, Give me an example.

5. What is your action if you found that your patient is at a high risk for developing VTE? Probes: If mention drugs, then ask what are the most common drugs that your prescribe in such a case and at what dose and for how long?

6. In your opinion, what are the advantages of assessing VTE risk for cancer patients? Prompt: the advantages for you as a physician, for the patient, and for the hospital that you work in

7. What type of help do you expect and want to get from pharmacists to aid in reducing VTE risk for cancer patients?

8. Are there any other comments?
