# Supplementary material for: Determining the perceptions and practices of oncologists regarding venous thromboembolism risk assessment in ambulatory cancer patients: A qualitative study
Source: PLoS One. 2025 Jan 6;20(1):e0316801. doi: 10.1371/journal.pone.0316801 (PMC11703059; doi:10.1371/journal.pone.0316801)
Supplement: S1 Table — (DOCX) [file pone.0316801.s002.docx]

**S1 Table: Characteristics of study participants**

| **Participant number** | **Gender** | **Age** | **Academic degree** | **Years of experience in oncology** |
| --- | --- | --- | --- | --- |
| P1 | Male | 39 | Iraqi board | 12 |
| P2 | Female | 36 | Iraqi board | 9 |
| P3 | Male | 60 | Diploma | 30 |
| P4 | Male | 55 | Diploma | 20 |
| P5 | Female | 35 | Iraqi board | 9 |
| P6 | Male | 60 | Diploma | 25 |
| P7 | Male | 37 | Diploma | 5 |
| P8 | Male | 40 | Iraqi board | 13 |
| P9 | Male | 39 | Diploma | 14 |
| P10 | Female | 35 | Iraqi board | 8 |
| P11 | Female | 33 | Iraqi board | 8 |
| P12 | Female | 40 | Iraqi board | 11 |
| P13 | Male | 37 | Iraqi board | 7 |
| P14 | Female | 41 | Iraqi board | 14 |
| P15 | Male | 41 | Iraqi board | 9 |
| P16 | Male | 39 | Diploma | 13 |
| P17 | Female | 38 | Iraqi board | 11 |
| P18 | Male | 39 | Iraqi board | 14 |
| P19 | Female | 38 | Diploma | 13 |
| P20 | Female | 36 | Iraqi board | 10 |
| P21 | Male | 38 | Iraqi board | 9 |
| P22 | Male | 38 | Iraqi board | 10 |
| P23 | Female | 40 | Egyptian board | 14 |
| P24 | Female | 34 | Iraqi board | 7 |
| P25 | Male | 40 | Iraqi board | 10 |
| P26 | Female | 36 | Iraqi board | 10 |
| P27 | Female | 35 | Iraqi board | 8 |
| P28 | Female | 38 | Iraqi board | 12 |
| P29 | Male | 60 | Iraqi board | 30 |
| P30 | Female | 36 | Iraqi board | 11 |
| P31 | Male | 50 | Egyptian board | 8 |
