# Supplementary material for: Determining the perceptions and practices of oncologists regarding venous thromboembolism risk assessment in ambulatory cancer patients: A qualitative study
Source: PLoS One. 2025 Jan 6;20(1):e0316801. doi: 10.1371/journal.pone.0316801 (PMC11703059; doi:10.1371/journal.pone.0316801)
Supplement: Attachment — Submitted filename: Review PLOS VTE Cancer.pdf [file pone.0316801.s003.pdf]

Review of "Venous Thromboembolism Risk Assessment in Cancer Patients: A Qualitative Study"

The abstract did introduced the significance of cancer-associated thrombosis (CAT) and the importance of thromboprophylaxis guidelines, while identifying a gap in the literature on guideline adherence. Clear objective was stated whereby the study aims to explore oncologists' perceptions and practices concerning VTE risk assessment among ambulatory cancer patients. The methods section did enhanced methodological rigor as it detailed the study design, time frame, and use of a semi-structured interview guide. Thematic analysis approach was also appropriate for qualitative data. The results section provides specific findings, such as the number of oncologists interviewed, their practices regarding VTE detection, risk assessment, and thromboprophylaxis. However, this section can be improve in its quantitative data presentation to be more consistent; for example, replacing "64% of participating oncologists emphasized that they didn't conduct VTE risk assessments" with "64% of oncologists reported not conducting VTE risk assessments." Integrating key findings more explicitly with the overall theme will also strengthen the results section. The conclusion summarizes poor adherence to guidelines is related to limited competence among oncologists, and suggests that pharmacists could enhance adherence. However, the conclusion could also add implications for practice and future research, such as the need for enhanced training and support for oncologists in VTE risk assessment and management.

Introduction

The introduction provides a rationale for conducting the study and included a comprehensive background on VTE in cancer patients and the current state of knowledge. The citations support the statements made, and the gap in knowledge regarding clinicians' adherence to guidelines is identified, however it may be relevant to identify and state who are considered as "high risk patients".

Methods

The study design is appropriate for the research question. Qualitative methods, including semi-structured interviews, are suitable for exploring oncologists' perceptions and practices. A concern that needs to be address is that the study mentions verbal informed consent, which is acceptable but it is advice that written consent will have more robust ethical compliance. The description of participant recruitment, setting, and data collection methods is direct, and authors provided validation of the interview guide by experts, which adds to the study's credibility. The coding and thematic analysis process is well-described, but it would be beneficial to mention how intercoder reliability was ensured.

Results

The results are presented clearly, with tables summarizing participant demographics and themes. The use of direct quotes from participants adds depth to the findings. Authors should consider that it may be more informative to have tables to summarize the results of the different cancers that were found to have VTE under the heading **"VTE among Cancer patients",** as well as the drugs administrated and the pharmacists role in managing VTE among cancer patients under the headings **"Dealing with cancer patients at high risk for**

VTE" and **"Pharmacists' role in the prevention of VTE**, so as to add clarity to the overall findings. Additionally, some quantitative data (e.g., percentages) in the qualitative study results section may benefit from further explanation to avoid confusion and linking the findings more explicitly to the research questions.

Discussion

The discussion effectively interprets the results in the context of existing literature. The identification of barriers to VTE risk assessment and the underutilization of guidelines are significant findings. The discussion could be improved from a deeper analysis of the implications of these findings for clinical practice and policy. The potential biases in the qualitative study, such as social desirability bias, are acknowledged, but strategies to mitigate these biases during data collection could be elaborated.

Conclusion

The conclusion summarizes the key findings and their implications. The suggestion that pharmacists can play a role in improving adherence to guidelines is practical and actionable, though further concrete suggestive practices for their role in managing VTE could be elaborated

General Comments

The study addresses a relevant and underexplored area in oncology practice. The focus on an Iraqi context adds originality and contributes to the global understanding of VTE risk assessment practices. Ethical approval and consent processes are mentioned, but more detail on how confidentiality and participant rights were protected would strengthen the ethical rigor. The manuscript is generally well-written, but minor grammatical errors and redundancies should be addressed to enhance readability.

Recommendation for Publication

The study focuses on a significant and overlooked aspect of oncology practice. The inclusion of an Iraqi context presents a distinctive viewpoint and raises the global understanding of VTE risk assessment practices. While the text briefly touches on ethical approval and consent processes, providing additional information on the measures taken to protect confidentiality and participant rights would enhance the overall ethical rigor of the study. The manuscript is well-written overall, but there are a few minor grammatical errors and redundancies that could be addressed to improve readability.

Suggested Revisions:

1. Provide details on written informed consent and measures to ensure ethical rigor.
2. Clarify the approach to ensuring intercoder reliability.
3. Address minor grammatical errors and improve overall readability.
4. Enhance the discussion of implications for clinical practice and policy.
