# Supplementary material for: Determining the perceptions and practices of oncologists regarding venous thromboembolism risk assessment in ambulatory cancer patients: A qualitative study
Source: PLoS One. 2025 Jan 6;20(1):e0316801. doi: 10.1371/journal.pone.0316801 (PMC11703059; doi:10.1371/journal.pone.0316801)
Supplement: Attachment — Submitted filename: Response to reviewer comments.docx [file pone.0316801.s004.docx]

**Dear reviewer 1,**

First of all, we want to express our gratitude for the time and effort you put into providing insightful feedback on our manuscript.

It is our pleasure to inform you that all of your suggestions and comments were carefully reviewed. We did our best to make nearly all of the suggested revisions in our manuscript. We believe that these changes have strengthened the overall quality and clarity of the manuscript, and we are confident that it now meets the standards of PLOS One.

We look forward to hear your feedback on the revised version and hope that it meets your expectations.

Thank you once again for your valuable comments and suggestions.

**Answers to reviewer 1 Comments**

1. In abstract (results): However, this section can be improve in its quantitative data presentation to be more consistent; for example, replacing "64% of participating oncologists emphasized that they didn't conduct VTE risk assessments" with "64% of oncologists reported not conducting VTE risk assessments."

**Response: A revision was done based on your valuable comment. Please refer to the highlights in the following statement.**

64% of participating oncologists reported that they did not conduct VTE risk assessments for their cancer patients.

2. Abstract: the conclusion could also add implications for practice and future research, such as the need for enhanced training and support for oncologists in VTE risk assessment and management.

**Response: A revision was done based on the given comment. Please refer to the highlighted statement.**

Educating and training oncologists about VTE risk assessment is recommended to enhance their practice in thromboprophlaxis.

3. Introduction: The citations support the statements made, and the gap in knowledge regarding clinicians' adherence to guidelines is identified, however it may be relevant to identify and state who are considered as "high risk patients".

Response: A new statement was added to identify who are considered as high risk patients. Please refer to the highlighted statement.

Therefore, current guidelines recommend VTE prevention for high-risk patients (who have about 7% risk to develop VTE) after assessing VTE and the bleeding risk of cancer patients [7,8].

4. Results: Authors should consider that it may be more informative to have tables to summarize the results of the different cancers that were found to have VTE under the heading "VTE among Cancer patients", as well as the drugs administrated

**Response: New tables (table 4 and table 5) were added. Please refer to the highlighted tables.**

Table 4: Types of cancer that reported to be associated with high risk for developing venous thromboembolism

| Type of cancer | No. of participants (%) |
| --- | --- |
| Gastric cancer | 17 (54.8) |
| Pancreatic cancer | 14 (45.2) |
| Breast cancer | 7 (22.6) |
| Lung cancer | 7 (22.6) |
| Colorectal cancer | 6 (19.4) |
| Gynecological cancer | 4 (12.9) |
| Pevlic cancer | 4 (12.9) |
| Prostate cancer | 2 (6.5) |
| Liver cancer | 2 (6.5) |
| Renal cancer | 1 (3.2) |
| Bladder cancer | 1 (3.2) |
| Lymphoma | 1 (3.2) |
| Brain cancer | 1 (3.2) |

*Some physician reported more than one type of cancer; therefore summation results in percentages more than 100%

Table 5: The most commonly prescribed anticoagulants for thromboprophylaxis among ambulatory cancer patients

| The most commonly prescribed anticoagulant | No. of oncologists prescribing the anticoagulant | Prescribed in a correct prophylactic dose n (%)^ |
| --- | --- | --- |
| Rivaroxaban | 9 | 1 (11.1) |
| Enoxaparin | 3 | 0 (0) |
| Aspirin | 2 | - # |
| Enoxaparin or Rivaroxaban | 4 | 1 (25)* |

^ Percentage was calculated based on the number of oncologists who prescribe the anticoagulant (not the whole study sample); # Dose not checked since Aspirin is not indicted for thromboprophylaxis; * Accuracy of reported dose was checked for both items.

5. The discussion could be improved from a deeper analysis of the implications of these findings for clinical practice and policy.

**Response: More details were added to discussion. Please refer to the following highlighted statements.**

This underutilization of validated scores for prediction of VTE among cancer patients raises a significant problem in the current clinical practice by most oncologists that must be addressed as soon as possible by policymakers in the Iraqi Ministry of Health (MOH). Meanwhile, such problem (underutilization of validated scores) is common in clinical practice not only in Iraq but also in the USA [31]. Therefore, increasing oncologists' awareness (through lectures and training workshops) about the importance of using the Khorana score for VTE risk assessment among ambulatory cancer patients is highly recommended. In addition, providing oncologists with Khorana score calculators can further enhance their clinical practice through adherence to VTE risk assessment guidelines by helping them to remember scores' parameters.

6. The potential biases in the qualitative study, such as social desirability bias, are acknowledged, but strategies to mitigate these biases during data collection could be elaborated.

**Response: More details were added. Please refer to the following highlighted statement.**

To reduce such type of bias, a strategy of building rapport with participants was used before the interview.

7. Ethical approval and consent processes are mentioned, but more detail on how confidentiality and participant rights were protected would strengthen the ethical rigor.

**Response: Details were added. Please refer to the following highlighted statement**

To achieve confidentiality, all participants were interviewed in a quiet area either in their office or in the consultation area while keeping a distance of at least 5 meters from other physicians or patients.

8. Provide details on written informed consent and measures to ensure ethical rigor.

**Response: Indeed, we obtained verbal informed consent from study participants, and this was already mentioned in our manuscript. However, we added more details about the issue of verbal informed consent. Please refer to the following highlighted statement.**

The current study was ethically approved by the ethical committee at the College of Pharmacy/University of Baghdad (approval number of RECAUBCP10620238). The ethical committee accepted waiving written informed consent due to cultural issues and potential fear from signing documents by most Iraqi individuals. Thus, it was requested that the authors obtain verbal informed consent from study participants.

9. Clarify the approach to ensuring intercoder reliability.

**Response: Details were added. Please refer to the following highlighted statements.**

The coding procedure was established by the first study author (MSc candidate in clinical pharmacy) and reviewed by the second study author (PhD in clinical pharmacy). Any discrepancy in coding was solved after negotiation and reaching consensus. The coding procedure was started by a thorough reading of each interview transcript. A codebook was developed to ensure consistent coding across interviews.

10. Address minor grammatical errors and improve overall readability.

**Response: The manuscript was checked manually for grammatical errors. All found errors were corrected.  Besides that we corrected all words which have no space between them. Then, the final manuscript version was further checked by subscribed grammarly application and all suggestions of the application were made when feasible.**

11. Enhance the discussion of implications for clinical practice and policy.

**Response: Details about this subject were added. Please refer to the following highlighted statements.**

This underutilization of validated scores for prediction of VTE among cancer patients raises a significant problem in the current clinical practice by most oncologists that must be addressed as soon as possible by policymakers in the Iraqi Ministry of Health (MOH). Meanwhile, such problem (underutilization of validated scores) is common in clinical practice not only in Iraq but also in the USA [31]. Therefore, increasing oncologists' awareness (through lectures and training workshops) about the importance of using the Khorana score for VTE risk assessment among ambulatory cancer patients is highly recommended. In addition, providing oncologists with Khorana score calculators can further enhance their clinical practice through adherence to VTE risk assessment guidelines by helping them to remember scores' parameters.

**Dear reviewer 2,**

First of all, we want to express our gratitude for the time and effort you put into providing insightful feedback on our manuscript.

It is our pleasure to inform you that all of your suggestions and comments were carefully reviewed. We did our best to make nearly all of the suggested revisions in our manuscript. We believe that these changes have strengthened the overall quality and clarity of the manuscript, and we are confident that it now meets the standards of PLOS One.

We look forward to hear your feedback on the revised version and hope that it meets your expectations.

Thank you once again for your valuable comments and suggestions.

**Answers to reviewer 2 Comments**

1. The title is misleading as it is a statement on the methodology or study design rather than the findings of the study.

**Response: The title was revised to be more comprehensive**

**Determining the Perceptions and Practices of Oncologists Regarding Venous Thromboembolism Risk Assessment in Ambulatory Cancer Patients: A Qualitative Study**

2. The author should run a spell check to correct words which have no space between them.

**Response: Done**

3. The data and questions in the data tool (Section B) are more related to practice rather than perception. There is much quantitative data and it is not clear how this was obtained. The study appears to have both quantitative and qualitative data. It will be good for the author to elaborate which results focus on practice and which data are for perception, how this was collected and analyzed.

**Response: Yes, you are right. Our questions are more related to practice. Details about which questions focus on practice and which for perception were added to method section. Details about the method for obtaining quantitative results were also added to methods. Please refer to the following highlighted statements,**

The validated interview guide consisted of eight questions, five (question 1 to 5) designed to assess oncologists practice in detecting and preventing VTE. All obtained answers regarding the type, dose, and duration of prophylactic anticoagulant were evaluated for appropriateness according to the recommendations in thromboprophylaxis guidelines [7,8]. Two questions were developed to assess oncologists' perceptions of the advantages of thromboprophylaxis and pharmacists' role in thromboprophylaxis. The last question was designed to capture any additional comments from participants.

**In addition, most obtained numbers in result section for example those who reported conducting VTE risk assessment were calculated manually based on responses from study participants. Adding numbers in qualitative studies has many advantages**

1. **It contributes to the internal generalizability of qualitative researchers’ claims**
2. **Quantitative data can help in identifying patterns that are not apparent simply from the unquantitized qualitative data**
3. **Quantitative data help to adequately present evidence for interpretations and to counter claims that researchers have simply cherry picked the data for instances that support these interpretations**

**Reference: Maxwell JA. Using Numbers in Qualitative Research. Qualitative Inquiry. 2010; 16(6) 475–482.DOI: 10.1177/1077800410364740**

4. In the results section on Dealing with Cancer patients with high risk of VTE: how did the author assess appropriateness of dosage? Was this part of the objective of the study?

**Response: Details were added to method section (please refer to the above). This part is indirectly present in study objectives that is related to exploring oncologists practice in VTE prevention (aim of study was slightly revised). Please refer to the highlighted change**

Therefore, the current study aimed to explore in depth the practices and perceptions of oncologists working in oncology centers in Baghdad, Iraq, regarding VTE, its risk assessment, and prevention among ambulatory cancer patients.

5. The conclusions are not supported by data from this study. The author concludes that limited competence as the main reason for poor compliance to guidelines for guidelines. How was compliance measured, and how did the author determine that there was poor compliance to thromboprophylaxis guidelines? Again, this as stated in the objective, was a study on perception and practice towards risk assessment for VTE.

Response: Conclusion was revised to be more compatible with study results. Please refer to the highlighted statements.

Cancer-associated thrombosis is somewhat prevalent among Iraqi cancer patients. Most participating oncologists reported benefits of VTE risk assessment to the patient by reducing morbidity and mortality and to the healthcare institution by lowering costs of treating VTE and its complications. Meanwhile, assessing VTE risk for ambulatory cancer patients is rarely conducted by oncologists working at Oncology centers in Baghdad, Iraq. The prophylactic anticoagulants were rarely prescribed in appropriate dose and/or duration for patients at high risk of VTE. Increasing the awareness of oncologists through lectures and training workshops about the importance of using the Khorana score for VTE risk assessment among ambulatory cancer patients can enhance their clinical practice. Pharmacists can help oncologists follow thromboprophylaxis guidelines by calculating VTE risk score and recommending a safe and effective dose of appropriate prophylactic anticoagulant.
