# Supplementary material for: Determining the perceptions and practices of oncologists regarding venous thromboembolism risk assessment in ambulatory cancer patients: A qualitative study
Source: PLoS One. 2025 Jan 6;20(1):e0316801. doi: 10.1371/journal.pone.0316801 (PMC11703059; doi:10.1371/journal.pone.0316801)
Supplement: Attachment — Submitted filename: Response to reviewer comments - 3rd revesion.docx [file pone.0316801.s005.docx]

**Dear reviewers,**

First of all, we want to express our gratitude for the time and effort you put into providing insightful feedback on our manuscript.

It is our pleasure to inform you that all of your suggestions and comments were carefully reviewed. We did our best to make nearly all of the suggested revisions in our manuscript. We believe that these changes have strengthened the overall quality and clarity of the manuscript, and we are confident that it now meets the standards of PLOS One.

We look forward to hear your feedback on the revised version and hope that it meets your expectations.

Thank you once again for your valuable comments and suggestions.

**Answers to reviewer 2 Comment**

The data presented in Table 4 is the number of oncologists who identified or selected that particular malignancy as being associated with VTE. The data does not represent the frequency of actual occurrence of VTE in patients with malignancy. The author may want to clarify this in the results.

**Response: A revision was done based on your valuable comment. We revise this to explicitly state that the data represent the number of oncologists who identified each type of cancer as being associated with VTE risk and not the frequency of VTE occurrences in these patient populations.**

**Please refer to the highlights in the following statement.**

In addition, the study participants reported that patients with certain types of cancer have a high risk of developing VTE (Table 4) such as gastric (n=17), pancreatic (n=14), breast (n=7), lung (n=7), colorectal (n=6), gynecological (n=4), pelvic (n=4), prostate (n=2), liver (n=2), kidney (n=1), bladder (n=1), lymphoma (n=1), and brain cancer (n=1). It is important to note that these findings reflect the observations of the participating oncologists among their patients in daily practice, rather than the actual incidence of VTE in these populations.

**Answers to reviewer 3 Comments**

1. The conclusion that CAT is prevalent in Iraqi cancer patients is inaccurate based on the data presented. The authors note that 71% of oncologists diagnose VTE in their patients. This suggests that most oncologists in the study have diagnosed VTE in cancer. Not that VTE is prevalent in cancer. To assess prevalence, you would need a denominator of all patients cared for by the 31 oncologists and calculate prevalence. Please revise.

**Response: A revision was done based on your valuable comment. Please refer to the highlights in the following statement.**

Conclusion: Cancer-associated thrombosis (CAT) is commonly diagnosed among Iraqi cancer patients. VTE risk assessment for ambulatory cancer patients is rarely conducted by oncologists working at Oncology centers in Baghdad, Iraq.

2. The statement that most prophylaxis prescriptions were inappropriate could use data for support in the abstract, especially given this is a conclusion of the study.

**Response: A new statement was added to abstract.** **Please refer to the highlighted statement.**

58% of oncologists reported that they prescribe thromboprophylaxis for high-risk patients; meanwhile, only 11% of them reported prescribing anticoagulants in a doses similar to that reported by thromboprophylaxis guidelines.

3. The introduction Paragraph 1-2 has jumping concepts. Recommended Modification for flow and clarity.

**Response: The introduction was revised according to your valuable comment.**

Venous thromboembolism (VTE) is a serious, potentially life-threatening condition and a major cause of mortality and morbidity [1][2]. Approximately 20% of all newly diagnosed cases of VTE are cancer patients [3], and an estimated 8% of cancer patients develop VTE within one year after diagnosis or progression of their malignancy [4,5]. Therefore, current guidelines recommend primary prophylaxis for VTE prevention in high-risk patients after assessing the competing risk of bleeding [6,7]. The prediction of VTE in cancer patients often involves the use of risk assessment models or scores (e.g., Khorana Score) based on the presence of certain clinical and laboratory factors [8].

Despite available evidence and existing guideline recommendations for VTE risk assessment of ambulatory cancer patients with primary thromboprophylaxis for identified high-risk patients, several studies found limited utilization of both risk assessment and prescription of primary prophylaxis in clinical practice [9,10,11]. However, several questions remain regarding assessing risk and prescribing prophylaxis. First, understanding clinician knowledge and understanding regarding guideline recommendations. Second, the frequency of VTE risk assessment and prescription of primary thromboprophylaxis for identified high-risk patients [10]. Therefore, the current study aimed to explore the practices and perceptions of oncologists working in Baghdad, Iraq, regarding cancer-associated VTE, its risk assessment, and prevention among ambulatory cancer patients.

4. Methods – change “validated by a panel of experts” to reviewed or endorsed by a panel of experts.

**Response: the statement was changed according to your valuable suggestion.**

The guide was reviewed by a panel of experts (Three academic clinical pharmacists with experience in qualitative research and one oncologist).

5. Table 2 can be moved to the supplement

Response: Table 2 were moved to Supporting information **(**supplement 2) (S2)

6. For the result section “VTE among Cancer Patients

Did the oncologists participating subspecialize? For example, were there oncologist that focus only on specific types of cancer? This will bias their answers.

b. For example, VTE is less common in patients with prostate cancer, but will be more common in pancreatic cancer etc. If you have a GU oncologist, they may be seeing VTE infrequently.

**Response: All participating oncologists hold a general medical or clinical oncology certificate and do not subspecialize in specific types of cancer. This means they treat a diverse range of cancer patients, which helps to provide a balanced perspective on the association between different cancers and the risk of VTE.**

7. Decrease use of the phrase “on the other hand”

**Response: We have carefully reviewed the manuscript and made adjustments to minimize its use**

8. For the abstract and results section stating that most patients are prescribed inappropriate dosing of prophylaxis, I recommend changing the result/interpretation to “of oncologists that prescribe primary thromboprophylaxis, most prescribe inappropriate doses compared to those recommended by guidelines or prior randomized controlled trials” (cite guidelines, AVERT, CASSINI).

**Response: It was revised. Please refer to the highlighted statement.**

**Of oncologists that prescribe primary thromboprophylaxis, most oncologists (n=10) prescribe inappropriate doses compared to those recommended by guidelines or prior randomized controlled trials [17,18]**

9. First line of the discussion is not supported by the data. The results stated that: 14 oncologists state that sometimes (not many) their patients develop and 8 stated that their patients commonly develop VTE. Sometimes does not = many. Also, this is not the main purpose of this study. This study is not to determine if VTE is common in cancer. It is to determine knowledge of VTE risk assessment and prophylaxis prescribing patterns, unless I misunderstood. Recommend deleting this sentence/conclusion throughout the paper.

**Response: the statement in the discussion was deleted according to your valuable comment.**

The result of the present study showed that most of the participating oncologists reported that many of their cancer patients develop VTE at some point in their life. Similar to the current finding, a large cohort study found that the incidence of VTE is high among cancer patients in California, USA [18].

10. Regarding type of cancer associated with VTE, again, this could be baised by the practice composition of the oncologist. I would not make this a key focus of the study. I recommend deleting all of paragraph 1 of the discussion. It is not supported by the study.

**Response: This information was obtained from physicians, and as we mentioned previously, we do not have a subspecialist in oncology. Therefore, we believe the data we collected is free from bias. For this reason, we chose to retain this information rather than remove it.**

**Additionally, we identified an unusual finding that could potentially serve as a foundation for future research, which is why we decided to keep this section. In response to the reviewer’s suggestion, however, we have removed the first part of the initial paragraph.**

**Furthermore, the current study findings (detection of VTE among patients with breast and prostate cancer) may raise attention to the possibility of a higher prevalence of VTE among Iraqi cancer patients than among those living in other countries. However, this assumption must be verified and explained by conducting well-designed clinical studies.**
